# Draft Rules Now Available

**Published:** 1921-07-02

**Authors:** 


### NURSE REGISTRATION IN SCOTLAND.

### Draft Rules Now Available.

Draft Rules for the formation, maintenance, and publication of the Register under the Nurses' Registration (Scotland) Act, with conditions as to fees, and the admission to the Register and its supplementary parts of existing nurses and nurses in training have now been issued. These Draft Rules are framed under Section III., 1 and 2, of the Act. Rules made under this Section do not come into operation unless and until they are approved by the Scottish Board of Health, and at least thirty days before making the Rules public notice of the fact and of the place where they may be obtained must be made. The Rules have also to be laid before each House of Parliament for twenty-one days before being passed.

The Various Registers.

Under the Act a General Register and three supplementary parts were constituted—*i.e.*, one for male nurses, one for mental nurses, and one for sick children's nurses. The Draft Rules provide for two additional supplementary Registers—*i.e.*, one for infectious disease nurses, and one for nurses trained in the nursing and care of mental defectives. A fee of one guinea is payable for registration in the general or any supplementary part of the Register, and 10s. 6d. for registration in any second or subsequent part; in addition, a yearly payment of 2s. 6d. for retention of the name in the Register must be made before September 30 in each year. The Register is to be made up to December 31 in each year, and if the Council so determine shall be published as soon as possible thereafter at a reasonable price. It shall contain the full names and addresses of all nurses registered under the Act, with the date of registration and the qualifications.

Conditions of Registration.

Within a period of two years after the date on which the Rules first come into operation an existing nurse (*i.e.*, one trained before November 1, 1919) may make application to be admitted to the Register or its supplementary parts on production of a certificate of character and certain other certificates as to knowledge and experience from at least two responsible persons, such as matrons of hospitals or registered medical practitioners under whom the applicant has worked. An applicant for admission to the general part of the Register must, in addition, supply a certificate of training from the Local Government Board for Scotland or from the Scottish Board of Health, *or* from a general hospital or institution recognised by the Council, *or* evidence that she has been for three years before November 1, 1919, *bona fide* engaged in practice as a nurse in attendance on the sick, and for at least the first of these three years in a hospital or institution for the treatment of the sick recognised by the Council. Where the applicant has been trained in a hospital not recognised by the Council she must supply such evidence of adequate knowledge and experience as the Council may consider satisfactory in each case, and also evidence that she has been *bona fide* engaged in practice as a nurse in attendance on the sick for three years before November 1, 1919. A certificate of not less than two years' training in a hospital or institution for the treatment of the sick recognised by the Council, and evidence of one year's training in a general hospital or institution having one or more resident medical officers recognised by the Council for training, will also qualify for admission to the general part of the Register, all such training being before November 1, 1919.

Pressure on our space prevents us dealing with the remaining Rules this week. Copies of the Draft Rules may be obtained from W. S. Farmer, Registrar General Nursing Council for Scotland, 13 Melville Street, Edinburgh.

